# An Improved Phase-Robust Configuration for Vibration Amplitude-Phase Extraction for Capacitive MEMS Gyroscopes

**DOI:** 10.3390/mi9070362

**Published:** 2018-07-22

**Authors:** Xudong Zheng, Siqi Liu, Yiyu Lin, Haibin Wu, Lai Teng, Zhonghe Jin

**Affiliations:** Micro-satellite Research Center, Zhejiang University, Hangzhou 310007, China; zhengxudong@zju.edu.cn (X.Z.); liusiqi@zju.edu.cn (S.L.); linyy@zju.edu.cn (Y.L.); haibinwu@zju.edu.cn (H.W.); jinzh@zju.edu.cn (Z.J.)

**Keywords:** microelectromechanical systems (MEMS) gyroscopes, phase delay variation, amplitude extraction, phase extraction, bias instability, quadrature error, long-time drift

## Abstract

This paper presents for the first time an improved algorithm for vibration amplitude-phase information extraction of capacitive microelectromechanical systems (MEMS) gyroscopes. Amplitude and phase information resulting from the improved algorithm is insensitive to the phase variation of an interface capacitance-voltage (CV) circuit, thus both long time drift of the gyroscope and bias instability have been improved. Experimental results show that both the phase and amplitude information extracted using this improved algorithm is insensitive to phase variation of CV circuit which is in accordance with theory. Bias instability using this improved configuration is 0.64°/h, which is improved two times more than the configuration using traditional double-side-band (DSB) demodulation configuration, and 4.3 times more than the configuration using single-side-band (SSB) demodulation, respectively. Allan deviation analysis shows that the slow varying drift term using D&S configuration is effectively reduced due to its robustness to CV phase variation compared to test results using DSB or SSB configuration.

## 1. Introduction

Microelectromechanical systems (MEMS) gyroscopes are highly sensitive to temperature. One of the reasons is that the Young’s Module of silicon varies with temperature which leads to dynamical parameter variation of MEMS gyroscopes. Besides, the temperature coefficient of the interface circuit also has an impact on the overall temperature dependence of MEMS gyroscopes. During the self-heating process, amplitude and phase response of the gyroscope interface circuit varies with temperature, resulting in slowly varying phase errors. These slowly varying phase errors contribute to gyroscope output drift. For MEMS gyroscopes utilizing control loops to regulate the drive amplitude at a set level and phase locked loop (PLL) to track resonance of gyroscope drive mode, variations in the interface circuit amplitude and phase response cause both the actual drive amplitude and frequency to deviate from its reference value, which is severe for gyroscope performance in the sense that the scale factor will fluctuate because it is directly determined by the multiplication of drive amplitude and frequency. Furthermore, the drive resonating frequency varies with temperature, which in turn affects the output of the interface capacitive circuit, resulting in lager errors for the acquired drive amplitude and phase information. As the amplitude response fluctuation can be calibrated by injecting a calibration signal in the drive loop to calibrate the variation of gyroscope drive dynamics and interface circuit [1], the phase variation of the interface circuit is often left undealt with. However, phase variation plays an important role in deterioration in gyroscope output drift and scale factor errors, even a small percentage of phase error could cause relatively quadrature error to leak into the angular rate channel, resulting in large zero offset errors and worse performance [2,3,4].

**Figure 1:**
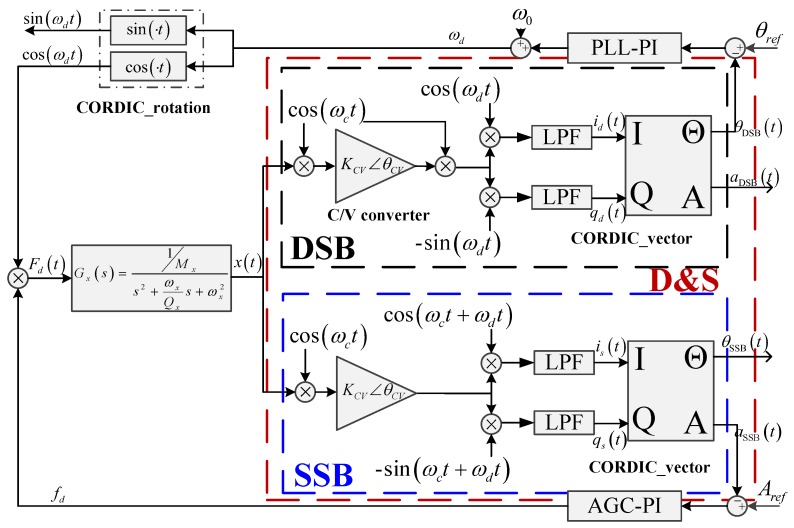
Illustration of double-side-band (DSB), single-side-band (SSB), and D&S signal processing configurations.

Most capacitive interface circuit modulates capacitive signal to a high-frequency carrier wave in order to reduce noise and avoid electrical coupling between gyroscope drive signal and capacitive output signal [5,6,7]. In order to gain the amplitude and phase information of gyroscope drive vibration, two demodulation configurations are often adopted. The first demodulation configuration utilizes the double-side-band demodulation technique to firstly move capacitive signal from high frequency to baseband, and then the baseband signal is demodulated for the second time with a pair of quadrature carrier signals of the same frequency to gain drive amplitude and phase information for the gyroscope [8,9,10], which is named DSB configuration for simplicity and illustrated in the top part of Figure 1. The second demodulation configuration directly demodulates one of the two side band signals with a pair of quadrature carrier signals of the same frequency to gain drive amplitude and phase information directly [11], which is named SSB configuration in this paper for simplicity and illustrated at the bottom part of Figure 1.

However, different amplitude and phase extraction configurations have different immunities to interface circuit errors mainly phase errors caused by temperature variation. Thus, the robustness of each information extraction configuration have different impacts on gyroscope performances.

In this paper, careful analysis and discussion of impacts of the two information extraction configurations on gyroscope performance are presented in Section 2. Then, we present an improved configuration (namely D&S configuration for simplicity) which combines the advantages of each of the two demodulation configurations and eliminates the side effects of the two configurations in Section 3. Benefiting from the immunity of the new configuration to slowly varying CV phase errors, gyroscope long time drift has been improved. Further, in Section 4, comparative experiments of the three configurations are conducted and compared with each other using the same MEMS gyroscope, which confirms the robustness of D&S configuration to CV phase errors and shows better long time drift performance for gyroscope using D&S configuration compared that using DSB or SSB configuration.

## 2. Theoretical Analysis

### 2.1. Analytical Study on Amplitude and Phase Information Extraction Using DSB and SSB Configurations

Neglecting the cross-coupling term from gyroscope sense mode to drive mode, the drive mode dynamics of the vibratory gyroscope is a typical second order mass-spring-damper system, and can be expressed as Equation (1). A lump-mass model is used here to illustrate how the phase delay variation of interface CV circuit acts on the performance of the gyroscope. For our gyroscope, the drive vibration is controlled at a reference value using an adaptive-gain-control loop despite of the details of the mechanical structure. For this reason, a lump-mass model is good enough for the analysis.

(1) $$\ddot{x}(t)+2\zeta_{x}\omega_{x}\dot{x}(t)+{\omega_{x}}^{2}x(t)=\frac{f_{d}(t)}{m_{x}}$$

where *m_x_*, *ζ_x_* and *ω_x_* represent mass, damping ratio and natural frequency of the drive mode, respectively. *f_d_*(*t*) is the applied electrostatic drive force. As shown in Figure 1 the driving displacement *x*(*t*) is converted to electrical signal via an interface capacitance-voltage (CV) circuit with gain *K_CV_* and phase delay *θ_CV_*. Assume *f_d_*(*t*) = *A_drive_* cos(*ω_d_t*), the displacement of drive mode can be expressed by Equation (2)

(2) $$x(t)=A_{dis}\cos(\omega_{d}t+\varphi )$$

where *A_dis_* and *φ* are the amplitude and phase of the drive displacement signal respectively. In order to eliminate the impact of the low-frequency noise of the interface circuit, the displacement *x*(*t*) is often modulated by a carrier signal with frequency *ω_c_* (*ω_c_ >> ω_d_*). To be more intuitive, the transfer function of the CV circuit is written briefly as *K_CV_*∠*θ_CV_*, with *K_CV_* representing the amplitude response and *θ_CV_* representing the phase response.

Using DSB configuration, there are two demodulation processes consisting of a first carrier demodulation using a reference signal (cos(*ω_c_t*)) to demodulate the capacitive signal back to base frequency *ω_d_*, and a second quadrature demodulation with a pair of quadrature reference signal (cos(*ω_d_t*) and sin(*ω_d_t*)) to gain vibration amplitude and phase information. Using SSB configuration, only one sideband, for example the sideband with frequency (*ω_c_ + ω_d_*), is directly demodulated with quadrature reference (cos(*ω_c_t + ω_d_t*) and sin(*ω_c_t + ω_d_t*)) to gain vibration amplitude and phase information. Neglecting high frequency components filtered out by lowpass filters, orthogonal demodulation output using DSB configuration and SSB configuration can be modelled using Equations (3) and (4) respectively.

(3) $$\left\{ \begin{array}{ll} i_{d}(t) & =x(t)\times \cos(\omega_{c}t)\times K_{CV}∠\theta_{CV}\times \cos(\omega_{c}t)\times \cos(\omega_{d}t)\times LPF\\ & \approx \frac{K_{CV}A_{dis}}{4}(\cos(\omega_{d}t+\varphi +\theta_{CV})+\cos(\omega_{d}t+\varphi -\theta_{CV}))\times \cos(\omega_{d}t)\\ & =\frac{K_{CV}A_{dis}}{4}\cos(\theta_{CV})\cos(\varphi )\\ q_{d}(t) & =x(t)\times \cos(\omega_{c}t)\times K_{CV}∠\theta_{CV}\times \cos(\omega_{c}t)\times \sin(\omega_{d}t)\times LPF\\ & \approx \frac{K_{CV}A_{dis}}{4}(\cos(\omega_{d}t+\varphi +\theta_{CV})+\cos(\omega_{d}t+\varphi -\theta_{CV}))\times \sin(\omega_{d}t)\\ & =\frac{K_{CV}A_{dis}}{4}\cos(\theta_{CV})\sin(\varphi ) \end{array}\right.$$

(4) $$\left\{ \begin{array}{ll} i_{s}(t) & =x(t)\times \cos(\omega_{c}t)\times \cos(\omega_{c}t+\omega_{d}t)\times K_{CV}∠\theta_{CV}\times LPF\\ & \approx \frac{K_{CV}A_{dis}}{2}(\cos(\omega_{c}t+\omega_{d}t+\varphi +\theta_{CV})+\cos(\omega_{c}t-\omega_{d}t-\varphi +\theta_{CV}))\times \cos(\omega_{c}t+\omega_{d}t)\\ & =\frac{K_{CV}A_{dis}}{4}\cos(\varphi +\theta_{CV})\\ q_{s}(t) & =x(t)\times \cos(\omega_{c}t)\times \sin(\omega_{c}t+\omega_{d}t)\times K_{CV}∠\theta_{CV}\times LPF\\ & \approx \frac{K_{CV}A_{dis}}{2}(\cos(\omega_{c}t+\omega_{d}t+\varphi +\theta_{CV})+\cos(\omega_{c}t-\omega_{d}t-\varphi +\theta_{CV}))\times \sin(\omega_{c}t+\omega_{d}t)\\ & =\frac{K_{CV}A_{dis}}{4}\sin(\varphi +\theta_{CV}) \end{array}\right.$$

where *i_d_*(*t*), *q_d_*(*t*) represent the orthogonal demodulation output signals using DSB configuration and *i_s_*(*t*), *q_s_*(*t*) are the orthogonal outputs using SSB configuration. Then the orthogonal output signal is processed by a CORDIC vector module to gain vibration amplitude and phase information, which are expressed by Equations (5) and (6) respectively. Amplitude and phase information using DSB configuration are represented by *A_d_*(*t*) and *θ_d_*(*t*), respectively. Similarly, *A_s_*(*t*) and *θ_s_*(*t*) are the derived results using SSB configuration.

(5) $$\left\{ \begin{array}{l} A_{d}(t)=\sqrt{{\left(i_{d}(t)\right)}^{2}+{\left(q_{d}(t)\right)}^{2}}=\frac{K_{CV}A_{dis}}{4}\cos(\theta_{CV})\\ \theta_{d}\left(t\right)=\arctan\left(\frac{q_{d}(t)}{i_{d}(t)}\right)=\varphi \end{array}\right.$$

(6) $$\left\{ \begin{array}{l} A_{s}(t)=\sqrt{{\left(i_{s}(t)\right)}^{2}+{\left(q_{s}(t)\right)}^{2}}=\frac{K_{CV}A_{dis}}{4}\\ \theta_{s}\left(t\right)=\arctan\left(\frac{q_{s}(t)}{i_{s}(t)}\right)=\varphi +\theta_{CV} \end{array}\right.$$

### 2.2. Influence of Interface Circuit Phase Variation θ_CV_ on Gyroscope Performance Using DSB and SSB

**Figure 2:**
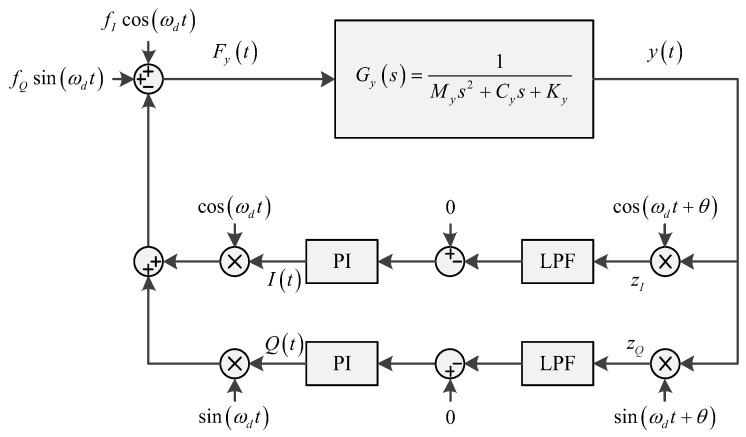
Sense-mode force-rebalanced control diagram.

The Coriolis acceleration caused by input angular rate is proportional to the drive-mode velocity, while the quadrature error in gyroscope sense mode is proportional to the drive-mode displacement. If force-rebalance control is implemented in the sensing mode which is shown in Figure 2, ideally the net force exerted on the sense proof mass is balanced, which can be expressed as Equation (7)

(7) $$\begin{array}{l} F_{I}\cos\left(\omega_{d}t\right)+F_{Q}\sin\left(\omega_{d}t\right)=2m_{y}\times \Omega \times {\left(x(t)\right)}^{\prime }+k_{xy}\times \left(x(t)\right)\\ =-2m_{y}\times \Omega \times \omega_{d}\times A_{dis}\sin\left(\omega_{d}t+\varphi \right)+k_{xy}\times A_{dis}\cos\left(\omega_{d}t+\varphi \right) \end{array}$$

*F_I_* and *F_Q_* each represent the amplitude of in-phase and quadrature force term to balance the Coriolis force and force, Ω is the input angular rate, *m_y_* is the sense mass, *k_xy_* is cross-coupling stiffness from driving mode to sensing mode. If the gyroscope driving mode is working in resonating mode, the displacement lags 90° compared to driving signal, thus *φ* is 90°. The equation can be simplified as

(8) $$F_{I}\cos\left(\omega_{d}t\right)+F_{Q}\sin\left(\omega_{d}t\right)=2m_{y}\times \Omega \times {\left(x(t)\right)}^{\prime }+k_{xy}\times \left(x(t)\right)=-2m_{y}\times \Omega \times \omega_{d}\times A_{dis}\cos\left(\omega_{d}t\right)-k_{xy}\times A_{dis}\sin\left(\omega_{d}t\right)$$

Ideally, the scale factor can be expressed as

(9) SFΩ_ideal=FIΩ=−2my×ωd×Adis

It is clear from Equations (5) and (6) that the amplitude information derived using DSB configuration and the phase information derived using SSB configuration are dependent on *θ_CV_*, while the phase information derived using DSB configuration and amplitude information derived using SSB configuration are independent of *θ_CV_*.

As a result, the phase delay of drive mode interface circuit *θ_CV_* has an impact on gyroscope performances.

#### 2.2.1. Using DSB Configuration

As illustrated in Figure 1, there are two loops in gyroscope drive mode, including an automatic gain control (AGC) loop which regulates the drive vibration amplitude to reference value *A_ref_* and a PLL loop to track the drive mode resonating frequency *ω_d_*.

If DSB configuration is adopted for the drive vibration amplitude and phase extraction, through Equation (3), the real vibration amplitude *a* can be expressed as

(10) $$A_{dis\_DSB}=\frac{4A_{ref}}{K_{CV}\cos(\theta_{CV})}$$

If we use *θ_CV_* = *θ_CV_CAL_* + *θ_CV_VAR_* to calibrate the phase of quadrature reference signal, where *θ_CV_CAL_* is the calibration phase for *θ_CV_* and *θ_CV_VAR_* is the variation of the calibration phase due to temperature variation, Equation (10) can be modified as

(11) $$A_{dis\_DSB}=\frac{4A_{ref}}{K_{CV}\cos(\theta_{CV\_VAR})}$$

Combining Equation (8) with Equation (11), the scale factor using DSB configuration can be expressed by Equation (12)

(12) SFΩ_DSB=FIΩ=−2my×ωd×4ArefKCVcos(θCV_VAR)

From Equation (12), even if the phase delay of CV interface circuit can be calibrated to a large extent using *θ_CV_CAL_*, *θ_CV_VAR_* due to temperature variation will still have an impact on the vibration amplitude, which causes scale factor error and zero output drift. Equation (12) can be expressed as Equation (13) to illustrate the impact of interface circuit phase variation on gyroscope scale factor.

(13) $$SF_{\Omega \_DSB}=\frac{SF_{\Omega \_ideal}}{\cos(\theta_{CV\_VAR})}$$

#### 2.2.2. Using SSB Configuration

In the case of using SSB demodulation configuration for the driving mode, since the driving dynamics introduces a constant phase delay of 90° at resonance, the reference value *θ_ref_* for PLL is set as 90° out-of-phase with respect to the drive signal. Because the vibration phase information extracted using SSB algorithm is dependent on the phase delay *θ_CV_* of interface CV circuit, again we use *θ_CV_CAL_* to calibrate $\theta_{CV}$, the driving-mode frequency cannot be locked into the exact mechanical resonating frequency due to *θ_CV_VAR_* caused by temperature variation.

This is expressed in Equation (14).

(14) $$\theta_{ref}=\varphi +\theta_{CV}=90^{\circ }+\theta_{CV\_CAL}=90^{\circ }+\theta_{CV}-\theta_{CV\_VAR}$$

Hence, the drive displacement can be rewritten as

(15) $$x\left(t\right)=A_{dis}\cos\left(\omega_{d}t+90-\theta_{CV\_VAR}\right)=-A_{dis}\sin(\omega_{d}t-\theta_{CV\_VAR})$$

Therefore, the equilibrium equation for the force-rebalanced sensing mode using SSB can be expressed as

(16) $$F_{I}\cos\left(\omega_{d}t\right)+F_{Q}\sin\left(\omega_{d}t\right)=2m_{y}\times \Omega \times {\left(x(t)\right)}^{\prime }+k_{xy}\times \left(x(t)\right)=-2m_{y}\times \Omega \times \omega_{d}\times A_{dis}\cos\left(\omega_{d}t-\theta_{CV\_VAR}\right)-k_{xy}\times A_{dis}\sin\left(\omega_{d}t-\theta_{CV\_VAR}\right)$$

Solving for Equation (16), we can get

(17) $$F_{I}=-2m_{y}\times \Omega \times \omega_{d}\times A_{dis}\cos\left(\theta_{CV\_VAR}\right)+k_{xy}\times A_{dis}\sin\left(\theta_{CV\_VAR}\right)$$

(18) $$F_{Q}=-2m_{y}\times \Omega \times \omega_{d}\times A_{dis}\sin\left(\theta_{CV\_VAR}\right)-k_{xy}\times A_{dis}\cos\left(\theta_{CV\_VAR}\right)$$

If SSB configuration is adopted for the drive vibration amplitude and phase extraction, through Equation (3), the real vibration amplitude *a* can be expressed as

(19) $$A_{dis\_SSB}=\frac{4A_{ref}}{K_{CV}}$$

and the scale factor using SSB configuration is

(20) SFΩ_SSB=FIΩ=−2my×ωd×4ArefKCVcos(θCV_VAR)+kxy×4ArefKCVsin(θCV_VAR)Ω=SFΩ_ideal×cos(θCV_VAR)+kxy×4ArefKCVsin(θCV_VAR)Ω

Scale factor using SSB configuration is affected by phase variation of CV interface circuit. As a result, leakage from the quadrature channel to angular rate channel will occur, which causes gyroscope zero drift and scale factor errors.

To conclude, drive amplitude and phase extraction using DSB configuration results an exact phase indication of gyroscope driving dynamics. Nevertheless, the vibration amplitude information is sensitive to CV circuit phase variation, which makes the gyroscope scale factor vary with *θ_CV_VAR_*.

The vibration amplitude obtained by SSB configuration is insensitive to the variation of *θ_CV_VAR_*. However, vibration phase obtained by SSB varies with *θ_CV_*, resulting quadrature error leakage into the angular rate measurement channel.

## 3. Robust Amplitude and Phase Extraction Configuration

An improved phase-robust demodulation configuration, namely D&S, which combines vibration phase information using DSB configuration and vibration amplitude information using SSB configuration, is proposed in this paper. The full algorithm of the D&S configuration is illustrated on Figure 1, which uses amplitude information extracted using SSB configuration and phase information extracted using DSB configuration. Thus, both the amplitude and phase information extracted using D&S configuration are immune to interface CV variations.

Since the amplitude of driving displacement is independent the CV circuit phase delay, drive amplitude can be expressed as

(21) $$A_{dis\_D\&S}=\frac{4A_{ref}}{K_{CV}}$$

As the phase information using D&S configuration is independent of interface circuit variation.

(22) $$\theta_{ref}=\varphi =90^{\circ }$$

The PLL loop tracks the exact resonance frequency of drive mode. Thus, there is no leakage from quadrature channel to angular rate measurement channel theoretically.

(23) $$F_{I}\cos\left(\omega_{d}t\right)+F_{Q}\sin\left(\omega_{d}t\right)=2m_{y}\times \Omega *{\left(x(t)\right)}^{\prime }+k_{xy}\times \left(x(t)\right)=-2m_{y}\times \Omega \times \omega_{d}\times A_{dis}\cos\left(\omega_{d}t\right)-k_{xy}\times A_{dis}\sin\left(\omega_{d}t\right)$$

(24) SFΩ_D&S=FIΩ=−2my×ωd×Adis=SFΩ_ideal

As both the drive amplitude and phase information extracted using this robust algorithm is insensitive to interface circuit phase variation, gyroscope scale factor is independent of CV phase delay and variation. Theoretically, gyroscopes using the D&S configuration should have better performance than those using DSB configuration or SSB configuration. We will discuss experimental results in Section 4.

## 4. Experimental Results

**Figure 3:**
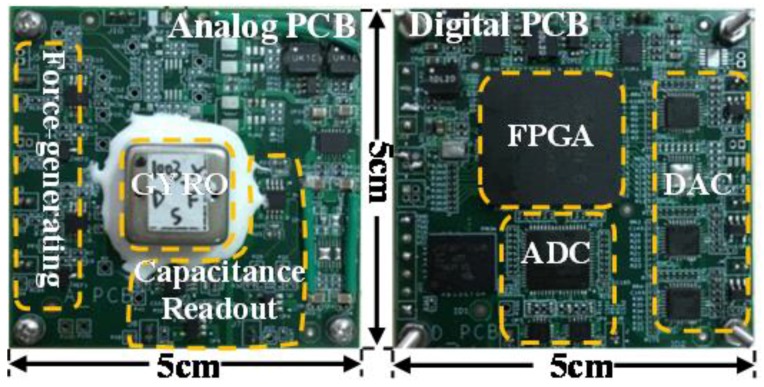
Evaluation platform for our gyroscope.

To compare test results of the gyroscope using D&S with that only using the DSB or SSB method, some comparative experiments are conducted using a field programmable gate array (FPGA) based digital platform on a vibratory micro-machined gyroscope, as shown in Figure 3. The analog interface circuit mainly consists of the MEMS gyroscope, the force-generating module and the capacitance-voltage module, while the digital circuit mainly comprises an FPGA device, analog-to-digital (A/D) converters and digital-to-analog (D/A) converters.

**Figure 4:**
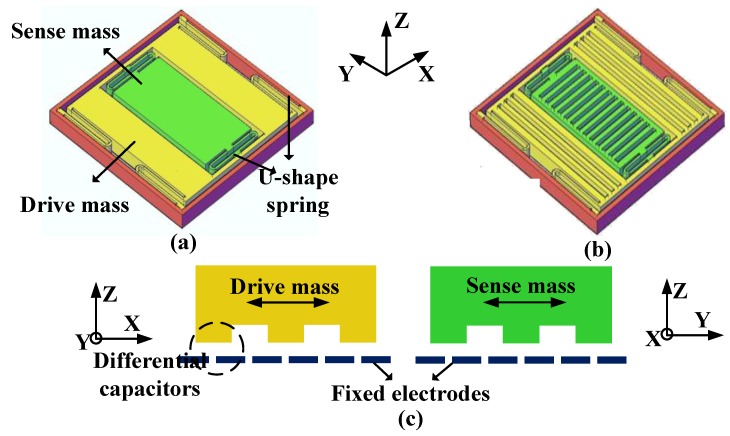
(**a**) Front view of the gyroscope mechanical structure (**b**) Back view showing the bottom of the silicon structure layer (**c**) Illustration of the bar electrodes.

The structure of the gyroscope is similar to that reported in [12], and shown in Figure 4. It is a capacitive gyroscope with an outer frame serving as drive mass and inner frame serving as sense mass. The drive mode (outer frame moving in the X direction) and the sense mode (inner frame moving in the Y direction) can be modeled using a lumped second order spring-damper-mass system. The drive mode is driven into resonation using electrostatic force which can be seen as a resonator. As illustrated in Figure 4c, we use a differential capacitor and push pull voltage pair to drive the gyroscope laterally. As the capacitance varies linearly with displacement in our gyroscope unlike parallel plate capacitors, the net force in the lateral direction is linear.

**Figure 5:**
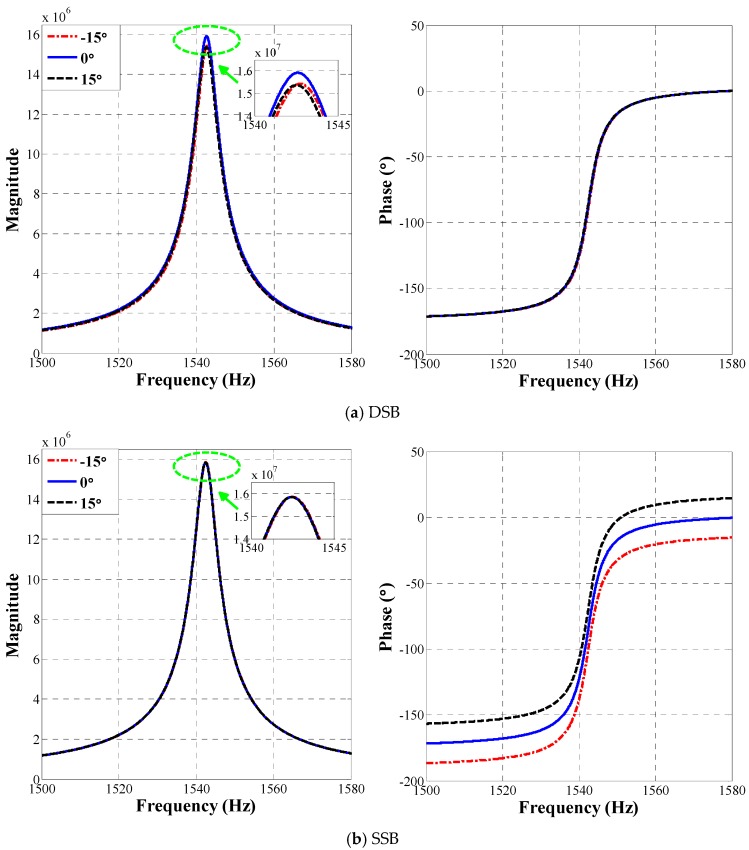

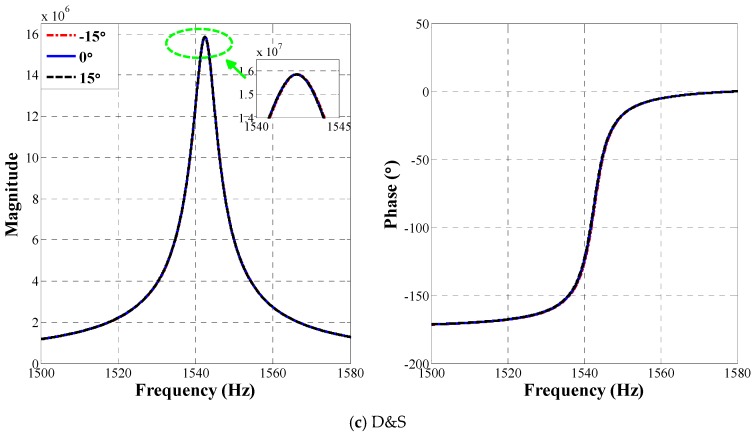
The measured frequency response of the drive mode, using (**a**) DSB configuration, (**b**) SSB configuration and (**c**) D&S configuration.

Firstly, frequency sweeping is applied to the gyroscope driving mode to gain magnitude-frequency and phase-frequency characteristic curves using each of the three configurations. In each system, we use a digital phase delay module to simulate phase delay variation of CV interface circuit; phase delay is set at −15°, 0°, +15°. Here, we choose a relatively large value of 15° difference in order to illustrate the robustness of D&S configuration. For MEMS gyroscopes, even a very small phase error in the order of 0.1° could cause severe quadrature leakage. The frequency of the drive signal sweeps from 1500 Hz to 1580 Hz altering 0.01 Hz at each 0.01 s. Figure 5 shows the magnitude and phase response of gyroscope using each of the three configurations. As Figure 5 shows, the drive mode resonating frequency is 1542.5 Hz with a quality factor of 220. It is important to note that both the magnitude-frequency and phase-frequency characteristics using D&S configuration are independent of the set phase delay, while the magnitude-frequency characteristic of DSB and phase-frequency characteristic of SSB are both sensitive to the set phase delay.

**Figure 6:**
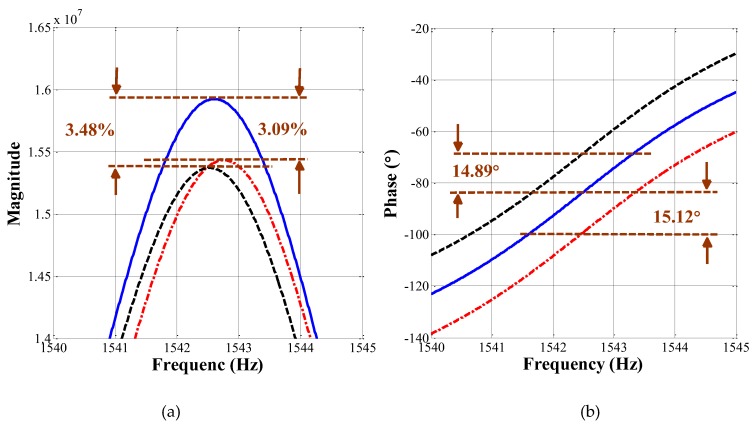
Enlarged frequency characteristic of the DSB (**a**) and SSB (**b**) systems in the vicinity of the drive-mode resonant frequency (1542.5 Hz).

Figure 6a enlarges the image in Figure 5a, which is one of the magnitude characteristics in the neighbourhood of the drive-mode resonant frequency (1542.5 Hz) in the DSB system. As the magnitude-frequency curve shows, the change in magnitude owing to the ±15° phase variations is calculated to be more than 3%. Figure 6b enlarges the image of the phase characteristics in the neighbourhood of the drive-mode resonant frequency (1542.5 Hz) in SSB system. As the phase-frequency curve shows, the change of resonant phase owing to the ±15° phase variations is calculated to be around 15°.

**Figure 7:**
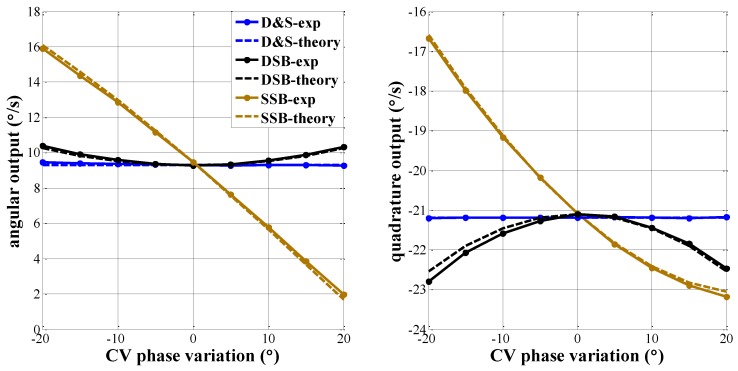
Robustness of sense-mode vibration against the phase delay of capacitance-voltage (CV) circuit in three systems.

Theoretical zero rate output and experimental test results for each of the three configurations with different phase variations are plotted in Figure 7. Both theory and experimental test results show that the zero output values using D&S configuration is robust to phase variation. Zero output of SSB is relatively large as a result of the leakage between the quadrature error and angular rate channel, which verifies the importance of phase accuracy in MEMS gyroscopes. A small phase error can cause a large output drift. Zero output of DSB is relatively modest because drive amplitude error has less impact on gyroscope output compared to phase error.

**Figure 8:**
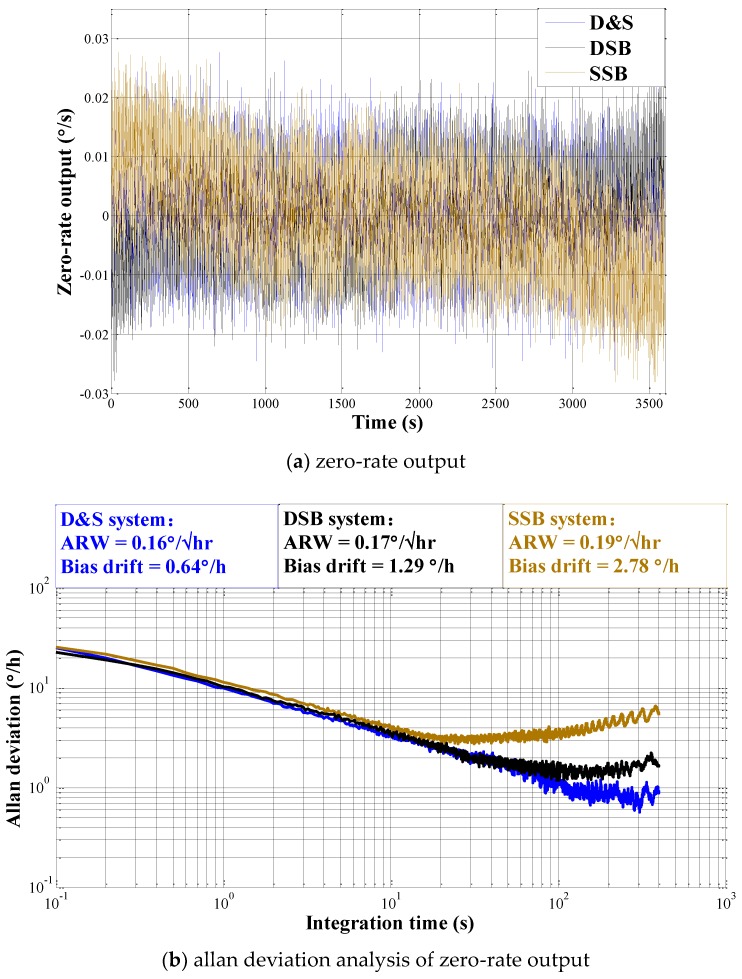
Zero rate output (**a**) and allan deviation (**b**) results of the measured gyroscope output.

Zero-rate output tests for the gyroscope using each of the three configurations is shown in Figure 8. The gyroscope output is collected at room temperature for 1 h with the sampling frequency of 10 Hz. The bias instability in D&S system is measured to be 0.64°/h (improved by 2 times than DSB and 4.3 times than SSB) at the longest integration time than in the other two systems. The angle random walk (ARW) in D&S system is measured to be 0.16°/h, which is similar to that of DSB and is slightly smaller than that of SSB. This makes sense because the amplitude information using D&S or DSB share the same signal processing architecture and information carried in both sidebands of the capacitive modulated signal are extracted while SSB only uses information from one sideband. Allan deviation analysis shows that the ramp at long averaging time is effectively reduced using D&S configuration compared to that using DSB or SSB, which is due to phase error robustness and thus the reduced quadrature error leakage into the angular rate channel using D&S configuration.

## 5. Conclusions

A modified information extraction configuration namely D&S configuration based on DSB and SSB configurations is proposed. Both theoretical analysis and experimental tests reveal the robustness of this information extraction configuration to the phase delay variation of the CV interface circuit in gyroscope drive mode. Comparative experiments using D&S, DSB and SSB configuration are conducted. Experimental results agree well with the theoretical analysis, showing that both the amplitude and phase information of the gyroscope drive mode are insensitive to the CV interface circuit phase variation. Thus quadrature leakage from gyroscope drive mode to sense mode is effectively reduced. As a result, a gyroscope using the D&S configuration has a better performance than that using DSB configuration or SSB configuration. Allan deviation analysis shows that slow varying drift term using D&S configuration is effectively reduced due to its robustness to CV phase variation resulting in less quadrature error leakage.
